# Dickson’s Essays on Pathology and Therapeutics

**Published:** 1845-12

**Authors:** 


### DICKSON'S ESSAYS ON PATHOLOGY AND THERAPEUTICS.

It is rare that we meet with a medical book, printed and published in Charleston, S. C. That, the title of which we have in part just written down, was lately sent to us through Messrs. Morton & Griswold, booksellers of this city. It is from the pen of the distinguished professor of the Institutes and Practice of Medicine in the Medical College of the State of South Carolina, and consists, as he tells us, of the substance of his course of lectures. Its publication would suggest the idea of his resignation, but no indication of that event appears in the book itself. Its title does not very accurately express its true character, as it is, strictly speaking, a work on the theory and practice; written, however, in the familiar style, in which it was spoken before the class. It is comprised in two neatly printed 8vo volumes. Of the first, about 200 pages are devoted to general ætiology and pathology; the remainder, after a brief general view of inflammation and fever, to particular forms of disease, not very carefully arranged. Being prepared for the instruction of students, it of course contains much with which the physician is familiar; nevertheless, Professor D. has incorporated so much of his own experience, and evinced, on the whole, so sound a judgment, that those who are already versed in the profession, may read it with advantage. We have not found time to do this in a thorough manner, but the perusal of several chapters, and a cursory examination of many more, have convinced us, that it abounds in sound doctrines, nowhere running into ultraism, and practical recommendations, in which great confidence may be reposed. To the students and physicians of the south it must prove a valuable compendium of practice; while those of the north may refer to it with advantage, as affording them data for comparison and contrast. We shall be happy to learn that it makes its way extensively among both classes.

